# Autonomic Dysregulation in Child Social Anxiety Disorder: An Experimental Design Using CBT Treatment

**DOI:** 10.1007/s10484-022-09548-0

**Published:** 2022-06-01

**Authors:** Julia Asbrand, Claus Vögele, Nina Heinrichs, Kai Nitschke, Brunna Tuschen-Caffier

**Affiliations:** 1 Department of Psychology, Albert-Ludwigs-University of Freiburg, Freiburg im Breisgau, Germany; 2 Department of Psychology, Humboldt-Universität Zu Berlin, Berlin, Germany; 3 Clinical Psychophysiology Laboratory (CLIPSLAB), Institute for Health and Behaviour, University of Luxembourg, Esch-sur-Alzette, Luxembourg; 4 Department of Psychology, University of Bremen, Bremen, Germany

**Keywords:** TSST, Sympathetic activity, Parasympathetic activity, Social stress, CBT, RCT

## Abstract

Models of social anxiety disorder (SAD) stress the relevance of physiological arousal. So far, limited research has been conducted in children with SAD in experimental stress designs. Thus, examining autonomic arousal, children with and without SAD completed a standardized social stressor (Trier Social Stress Test for Children-C; TSST-C). Pre-existing differences to healthy controls (HC) were expected to decrease after receiving cognitive behavior therapy (CBT). Children with SAD (n = 64) and HC children (n = 55) completed a TSST-C. Children with SAD participated in a second TSST-C after either cognitive-behavioral treatment or a waitlist-control period (WLC). As expected, children with SAD showed blunted heart rate reactivity compared to HC children. Further, children with SAD had elevated levels of tonic sympathetic arousal as indexed by skin conductance level compared to HC. Children with SAD showed lower parasympathetic arousal during the baseline compared to HC. Children receiving treatment did not differ from children in the WLC condition in a repeated social stress test. Psychophysiological differences between children with SAD and HC children could be confirmed as indicated by previous research. The lack of physiological effects of the intervention as an experimental manipulation might be related to slower changes in physiology compared to e.g. cognition.

**Supplementary Information:**

The online version of this article contains supplementary material available 10.1007/s10484-022-09548-0.

## Introduction

Social anxiety disorder (SAD) is one of the most common mental disorders in children and youth (lifetime prevalence 9%; Burstein et al., 2011). It entails the persistent fear of rejection by others and humiliation, leading to impairment of affected children due to fewer friendships, poorer academic success, and the development of comorbid disorders (Rao et al., 2007). While cognitive models explain the cognitive nature of SAD (Clark & Wells, 1995), the physiological qualities have yet to be clarified. These cognitive models propose that individuals with SAD experience heightened physiological reactivity in response to social evaluative stress (Clark & Wells, 1995). Reactivity includes bodily changes in response to stressful stimuli or events as for example indicated by an elevated heart rate. In contrast, physiological models assume that anxious individuals are not characterized by higher autonomic reactivity but rather by chronically elevated autonomic arousal during rest and lower autonomic reactivity to feared situations, leading to restricted autonomic flexibility in anxious individuals (e.g., Friedman, 2007; Porges, 2007; Thayer & Lane, 2000). Acute physiological arousal is partly regulated by the autonomic nervous system (ANS), consisting of the sympathetic (SNS) and parasympathetic (PNS) branches. The ANS allows for fast and flexible reactions to stressful situations, such as fight-or-flight responses, by increasing SNS activation and/or decreasing PNS activation. Subsequent relaxation in turn is mediated by decreased SNS and/or increased PNS activity (Berntson et al., 1994). Typical indicators of ANS activity include heart rate (HR), skin conductance level (SCL), and heart rate variability (HRV; e.g., Kreibig, 2010). Social stress in the laboratory can be measured in a standardized manner by using the Trier Social Stress Test for Children (TSST-C; (Buske-Kirschbaum et al., 1997). The TSST-C has been deemed a valid psychosocial stressor for inducing physiological stress responses in children and adolescents (Seddon et al., 2020). Most importantly, it does not only induce stress in children and adolescents with clinical levels of anxiety but in healthy controls as well (e.g., (Schmitz et al., 2011) and, thus, allows the comparison of not only baseline levels, but also social-evaluative stress and thus reactivity, and a post-recovery phase. Recovery here relates to the subsequent return to baseline stress levels.

### Autonomic Response in Children with SAD

In children with SAD, results on autonomic functioning are inconsistent. In line with others (e.g., Miers et al., 2011), Anderson and Hope (2009) found no differences between youths with SAD and healthy controls in physiological reactivity to a social performance task. In contrast, other studies (Matthews et al., 1986; Schmidt et al., 1999) have reported elevated finger pulse reactivity in subclinical socially anxious children during a read-aloud task. Three previous studies (Krämer et al., 2012; Schmitz et al., 2011, 2013) including children with SAD and subclinical socially anxious samples found higher SNS activity during baseline and blunted autonomic stress task responding. This so-called restricted autonomic flexibility (e.g., Thayer & Lane, 2000) indicates an autonomic stress response with slow adaptation from baseline to stress to recovery. High flexibility may provide the autonomic basis for responsivity (strong response), energy efficient (swift recovery) and adaptive coping with emotional and physical stressors as claimed by several reviews (Beauchaine, 2001; Porges, 2007; Porges et al., 1996; Thayer & Lane, 2000). Importantly, these swift autonomic adjustments are thought to be mediated primarily by the nervus vagus, assessed via HRV. HRV describes the variation in the time interval of between heartbeats. It is measured by the variation in the beat-to-beat interval and entails the direct relation between neural activity and autonomic activity (Mulcahy et al., 2019). Additionally, HRV might be a quantification of the extent to which adaptive regulation can produce flexible control over peripheral processes (Thayer et al., 2012; Williams et al., 2015). From a clinical point of view, in a meta-analysis lower HRV at resting has been associated with higher levels of anxiety during both resting and orthostatic stress (Paniccia et al., 2017). Further, several studies have documented the importance of autonomic flexibility in the development of anxiety disorders (Greaves-Lord et al., 2010; Monk et al., 2001; Rozenman et al., 2017; Yeragani et al., 2001) with only few contradictory findings (e.g., Alkozei et al., 2015). For example, Rozenman et al. (2017) showed that youths with a primary anxiety disorder demonstrated elevated HR and suppressed HRV at the beginning of an error feedback task and during the recovery period compared to a healthy control group. In a review of 28 studies on psychophysiological differences in childhood social anxiety (Siess et al., 2014), the authors conclude that autonomic reactivity alterations in SAD samples most likely occur under two conditions: during and before intensive laboratory social stressors and when using broad assessment of both SNS and PNS parameters, capturing even subtle and mild alterations in ANS activity. Interestingly, the blunted reactivity modulated by the PNS seems to be specific to social stress, as a study conducted in a familiar environment using a physiological stress task (i.e. inducing bodily stress but no social or emotional stress, e.g. orthostatic stress and climbing a step) did not show differences between children (aged 9 to 13 years) with and without SAD (Asbrand et al., 2017). Previous results used a variety of laboratory challenges (e.g. speech tasks, (Alkozei et al., 2014; Anderson & Hope, 2009), math tasks (Rozenman et al., 2017), orthostatic tests (Asbrand et al., 2017; Greaves-Lord et al., 2010), sometimes rather small samples (e.g., Yeragani et al., 2001) and samples with subclinical and clinical levels of social anxiety (e.g., high social anxiety (Schmitz et al., 2013), SAD (Asbrand et al., 2017; Schmitz et al., 2011)). To overcome these shortcomings of previous research, we, therefore, used an ecologically valid but highly standardized social stress task, the Trier Social Stress Test for Children (Buske-Kirschbaum et al., 1997) with a large sample of children with diagnosed SAD.

### Mechanisms of the Stress Response

To address the question of causal effects, the repeated presentation of the TSST-C using an experimental manipulation is necessary. In adults, only few studies have used repeated TSST assessments (cf. Allen et al., 2014), indicating a lack of habituation in HR. The undiminished HR reactivity to the TSST has been associated with continued elevated levels of general arousal due to the performance character of the TSST, while other parameters habituate (e.g. hypothalamus–pituitary–adrenal axis) if the repetition occurs after a few days. Even fewer studies have used treatment as an experimental approach to examine changes in reactivity over time between two TSSTs in adult samples (Britton, et al., 2012; Hoge et al., 2018): Britton et al. (2012) found decreased emotional reactivity to the TSST after using mindfulness based cognitive therapy for adults with remitted depression. Hoge et al. (2018) included physiological parameters and found reduced levels of cortisol and inflammatory cytokines in adult patients with generalized anxiety disorder after mindfulness meditation training. No experimental research has yet been conducted with children, much less with children with SAD to investigate the physiological mechanisms which may contribute to or are associated with this condition. In addition, compared to research on hormonal stress responses to the TSST, much less research has been conducted to examine the role of the ANS as indexed by cardiovascular and electrodermal responses to social stress (Allen et al., 2017). Thus, to understand the potential value of physiological arousal in child SAD etiology, a repeated measure of arousal in a stress situation is necessary including an experimental manipulation of change (e.g., a cognitive behavioral treatment [CBT] as gold standard in the treatment of anxiety disorders (Higa-McMillan et al., 2016)). Previous studies have shown that CBT is indeed able to change symptoms of anxiety (Chu & Harrison, 2007). Most interestingly, findings from the current sample have shown a CBT induced change in social anxiety symptoms (Asbrand et al., 2020) and cognitions (Asbrand et al., 2019). However, research on effects of CBT aside from core symptoms such as anxiety or cognitions is scarce.

### The Current Study

This study investigated psychophysiological processes deemed to be relevant for the etiology and maintenance of SAD in children. First, we exposed children with and without SAD to a relevant social stress stimulus. We expected a similar pattern as shown in previous, non-clinical studies (Schmitz et al., 2013), i.e. higher baseline levels of HR and lower levels of HRV as well as a tonic hyper-activity of electrodermal activity (EDA) as indicated by SCL in children with SAD compared to healthy control children (Asbrand et al., 2017; Siess et al., 2014), but no reactivity differences. Second, in an attempt to further elucidate the role of ANS activation patterns in SAD, we used CBT to experimentally induce changes in responses to social stress in children with SAD. We expected pre-existing differences (compared to HC) in HR, HRV during baseline and throughout social stress to decrease in the group receiving CBT. More specifically, we expected sympathetic parameters to recover more quickly in children with SAD in the CBT group. As there are no results on repeated TSST-C measures in children with social anxiety disorders, no clear assumptions could be made for the waitlist control (WLC) group concerning habituation, stability of effects, or sensitization.

## Method

### General Trial Design

The project was designed as a cross-sectional study (including children with and without SAD) followed by a randomized controlled trial using CBT as an experimental manipulation (for children with SAD only). The project was designed based on cognitive models of SAD and aimed at comparing affective, cognitive and physiological processes in social anxiety. Eligibility criteria were registered with the German Research Foundation (TU 78/5–2, HE 3342/4–2) prior to recruitment and not changed during the study. The entire project consisted of experimental studies related to research questions of visual attention allocation or psychophysiological processes under (social) stress and it also aimed to measure treatment success by including several outcome variables (state anxiety, negative cognitions, physiological arousal [cortisol, sympathetic arousal, heart rate], perception of and worry about physiological symptoms, perception of academic performance, negative post-event processing, parental cognitions, parental fear of negative child evaluation, and related treatment outcome predictions). We reported the majority of the a priori defined outcome variables in three papers.^1^

The current study now includes a detailed heart rate analysis, which had been classified a priori as primary outcome variable (i.e., improvement of sympathetic arousal after CBT). Additional measures of ANS activity were included here post-hoc to allow a more specific focus on psychophysiological processes in SAD independent of intervention efficacy considerations but were not a prioi defined as outcome measures. Concerning CBT effects, variables were not a priori defined as primary outcome measures but were based on an exploratory approach. As there are no published reports on children with SAD using a laboratory stressor and psychophysiological measures as stress parameters, the sample size was determined based on physiological models, and a power analysis was conducted for two groups and seven measurements (1-β = 0.95, f = 0.15, α = 0.05, r_between measures_ = 0.0) and set at N = 98. As the current study was part of a larger research project requiring a larger sample size of at least N = 110, all children were included to increase power. As the current study is part of a larger project of which other results have been published previously, the following method section is based on these reports (Asbrand et al., 2019, 2020).

### Participants

**Fig. 1:**
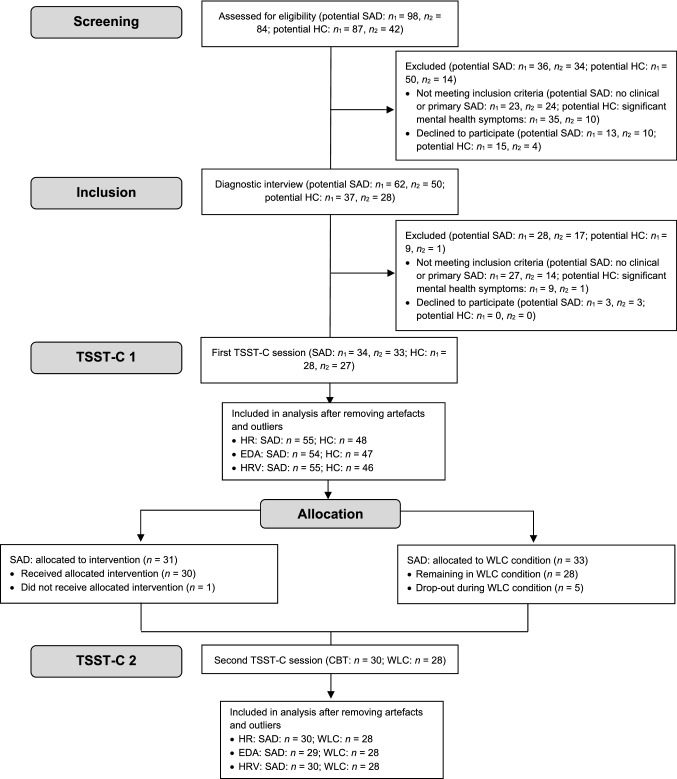
Flowchart of study participants (with a focus on psychophysiological variables). *n*_*1*_ Center 1, *n*_*2*_ Center 2; *CBT* cognitive behavioral therapy, *EDA* electrodermal activity, *HC* healthy control, *HR* heart rate, *HRV* heart rate variability, *SAD* social anxiety disorder, *TSST-C* Trier Social Stress Test for Children; WLC = waitlist control

As reported previously (Asbrand et al., 2019, 2020), parents of anxious and non-anxious children (9 to 13 years) were approached through advertisements in schools, medical facilities and newspaper articles in two midsized German cities from January 2012 to November 2013 until the targeted sample size had been reached (for an overview see Fig. 1). As compensation for participation in the laboratory study, parents received 35€, and children 25€ in vouchers. An independent ethics committee (ethics committee of the German Society for Psychology [DGPs]) granted ethical approval for this study. Participating children and their caregivers gave oral and written informed consent.

Inclusion criteria for children in the SAD group consisted of SAD as primary diagnosis. Inclusion criteria for children in the HC group consisted of no current or lifetime diagnosis of a mental disorder. Exclusion criteria included health problems or medication, which could have interfered with psychophysiological assessment (e.g., asthma, cardiac arrhythmia, and methylphenidate). Demographics and psychometric measures are reported in Table 1. The groups did not differ in age, type of school or any of the disorder-specific measures. Social Phobia and Anxiety Inventory for Children (SPAI-C) scores exceeded the suggested cut-off for clinically relevant SAD.

**Table 1:** Participant Characteristics SAD vs. HC This table has been adapted from Asbrand et al. (2019)

Characteristic	Group		Statistics
	Social anxiety disorder	Healthy controls	
n^a^	64	55	
Mean age (SD), in years	11.3 (1.4)	11.3 (1.4)	t_(117)_ = 0.06, n.s
Female	63.6%	60.0%	χ^2^_(1)_ = 0.17, n.s
Mean SPAI-C (SD)	23.3 (9.03)	4.2 (5.4)	t_(117)_ = -13.71***
Net income (per month)			χ^2^_(7)_ = 11.42, n.s
n.a	0%	1.3%	
< €1000	0%	5.9%	
€1001–1500	1.9%	7.4%	
€1501–2000	11.1%	8.8%	
€2001–3000	35.2%	32.4%	
€3001–4000	14.8%	16.2%	
€4001–5000	14.8%	20.6%	
> €5000	22.2%	7.4%	
Mean (SD) state anxiety during TSST-C (before treatment)	6.6 (2.8)	4.5 (2.9)	t_(117)_ = 4.05***

*SPAI-C* Social Phobia and Anxiety Inventory for Children, *TSST-C* Trier Social Stress Test for Children

***p ≤ .001, n.s. = not significant (p > .05)

^a^Sample sizes differ as not all questionnaires were completed correctly

Participants with SAD were block randomized to either CBT or WLC. Randomization for each research center was conducted in a concealed fashion by the other center, based on subject codes, as soon as there were a sufficient number of participants for one experimental and one waitlist-control group. Children in the CBT and WLC condition did not differ in sociodemographic or psychopathological variables (see Table 2). Characteristics of the sample throughout the project are shown in the flowchart (Fig. 1).

**Table 2:** Participant Characteristics CBT vs. WLC before CBT. This table has been adapted from Asbrand et al. (2019)

Characteristic	Group		Statistics
	Treatment group (CBT)	Waitlist Control Group (WLC)	
n^a^	31	33	
Mean age (SD), in years	11.5 (1.4)	11.2 (1.3)	t_(62)_ = 0.78, n.s
Female	51.6%	67.6%	χ^2^_(2)_ = 1.88, n.s
Comorbid diagnoses	41.9%	45.9%	χ^2^_(2)_ = 1.66, n.s
Mean SPAI-C (SD)	11.8 (7.3)	12.1 (7.1)	t_(62)_ = 0.18, n.s
Net income (per month)			χ^2^_(7)_ = 6.65, n.s
n.a	3.2%	0%	
< €1,000	6.5%	5.6%	
€1,001–1,500	9.7%	5.6%	
€1,501–2,000	6.5%	8.3%	
€2,001–3,000	41.9%	23.7%	
€3,001–4,000	16.1%	16.7%	
€4,001–5,000	9.7%	30.6%	
> €5,000	6.5%	8.3%	
Mean (SD) state anxiety during TSST-C (before treatment)	6.7 (2.9)	6.6 (2.8)	t_(62)_ = 0.10, n.s
Mean (SD) state anxiety during TSST-C (after treatment)	6.7 (2.8)	5.5 (3.7)	t_(55)_ = 1.33, n.s

*CDI* Child Depression Inventory, *SPAI-C* Social Phobia and Anxiety Inventory for Children, *TSST-C* Trier Social Stress Test for Children, *n.a.* not available

^a^Sample sizes differ as not all questionnaires were completed correctly

n.s. = not significant (p > .05)

### Treatment

Group CBT consisted of a disorder-specific intervention following a published manual (Tuschen-Caffier et al., 2009). It consists of typical CBT components psychoeducation, cognitive restructuring, exposure sessions, and self-management. Each of the weekly 12 session took 100 min and was delivered by trained clinical psychologists and psychotherapists. The treatment led to a significantly stronger decrease in symptom severity in the CBT group when compared to the children in the WLC group (for more details see [reference excluded to ensure authors’ anonymity]). The intervention was not specifically tailored to address psychophysiological levels. However, as usually expected with CBT treatments, exposure and also cognitive change are assumed to also be associated with changes in bodily symptoms. We therefore conceptualized the intervention as an experimental manipulation for these physiological processes.

### Procedure

**Fig. 2:**
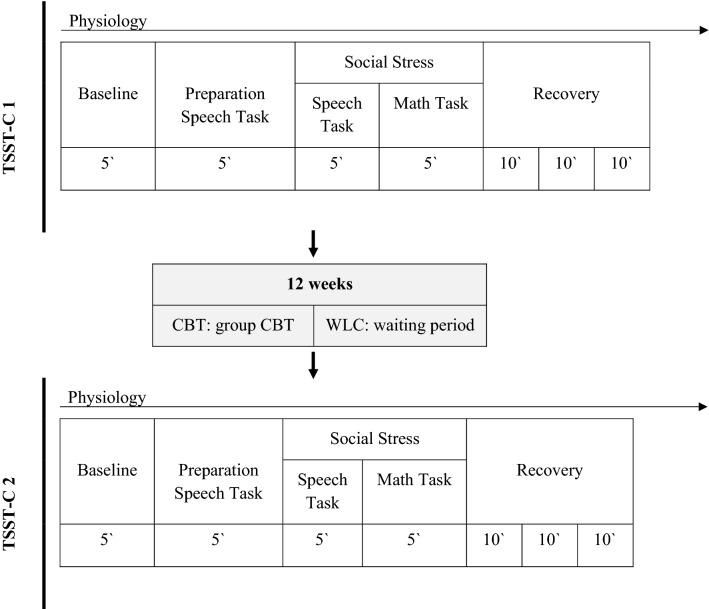
Procedure

At first, children and parents each completed an individual structured clinical interview (Diagnostic Interview for Mental Disorders in Children and Adolescents (Kinder-DIPS; (Schneider et al., 2009)), supervised by an experienced clinical psychologists. The Kinder-DIPS is a validated instrument for child mental disorders (Schneider et al., 2009). Families further reported sociodemographic data, anxiety symptoms, and general psychopathology in questionnaires. Afterwards, children with and without SAD participated in the first laboratory session. It consisted of a speech and a math task divided into 7 phases (Trier Social Stress Test for Children; TSST-C; Buske-Kirschbaum et al., 1997). In the speech task, children were asked to continue narrating a story in front of two observers after listening to the beginning of the story (stories see Supplement S1). In the following mental arithmetic task, children were asked to serially subtract the number 7 from 758 (9- to 11-year-olds) or the number 13 from 1,023 (12- to 13-year-olds) as fast and as accurately as possible and provide a verbal answer in front of the two observers. Both observers were instructed and trained to give neutral verbal and nonverbal feedback. Psychophysiological arousal was assessed throughout the TSST-C (see Fig. 2). After participating in a 12-week CBT program (CBT group) or waiting without treatment (WLC group), children with SAD were tested again using the identical experimental protocol as in the first laboratory session. Following the protocol of the original TSST-C (Buske-Kirschbaum et al., 1997), the speech task was changed to a different story that was evaluated as being similarly interesting and difficult in a pre-evaluation. This story was pre-evaluated with children from the same age group to achieve a similar level of excitement and difficulty. The math task was changed to a different start number (+ 10).

### Psychometric Measures

#### Social Phobia and Anxiety Inventory for Children (SPAI-C)

We used the SPAI-C (Beidel et al., 2001) as a continuous measure of social anxiety. The SPAI-C assesses behavioral characteristics, cognitions, and physiological symptoms of SAD by providing a range of potentially anxiety-producing situations (e.g., reading aloud, performing in a play, eating in the school cafeteria). Validity and reliability have been confirmed in both the original sample (Beidel et al., 2001) and a German sample (Melfsen et al., 2011). The internal consistency of the SPAI-C in the current sample was excellent (α = 0.98).

### Psychophysiological Measures

Electrodermal and cardiovascular measures including heart rate were assessed at 400 Hz using the Varioport system (Becker Meditec, Karlsruhe, Germany). Data inspection and artefact rejection were conducted offline using ANSLAB (Blechert et al., 2016). For the ECG, cardiac inter-beat interval (IBI), calculated as the interval in milliseconds between successive R-waves, was extracted. For illustrative purposes the IBI was converted to HR (in beats per minute [bpm]) for tables and figures but all statistical analyses where based on IBI values (Quigley & Berntson, 1996). HR has been reported before in a different context including more general analyses (Asbrand et al., 2020). Electrodermal activity (EDA), reflecting electrodermal sympathetic activity (Boucsein, 2011) was assessed by placing two electrodes on the middle phalanx of the middle and ring fingers of the left hand using 11-mm inner diameter Ag/AgCl electrodes filled with isotonic electrode paste (TD-245, Med Associates, Inc., St. Albans, Vermont). As a parameter of EDA, skin conductance level (SCL) in μmhos was used.

HRV was used as an index parasympathetic activity. HRV was calculated using complex demodulation (CDM; Hayano et al., 1994; Wilhelm, Grossman, & Roth, 2005). It provides time-dependent changes in an instantaneous amplitude of the oscillatory component within a given frequency range. Therefore, CDM is suitable for the assessment of HRV during acute mental or physiological stress, when the assumption of stationarity for spectral analysis is violated. For assessing the magnitude of HRV, we demodulated the amplitude of the IBI oscillation in a high frequency band (0.15–0.50 Hz), representing vagal control (Mezzacappa et al., 1997). To confirm the validity of the measure, we additionally used the Root Mean Square of the Successive Differences (RMSSD), which is a further valid marker for HRV (Ciccone et al., 2017).

### Statistical Analysis^2^

For the separate analyses of all physiological measures, the open-source statistics software R was applied (R Core Team, 2013) using the mixed-model packages lme4 (Bates et al., 2014), lmerTest (Kuznetsova et al., 2014), emmeans (Lenth, 2020) and MuMIn (Bartoń, 2019). For the before treatment analysis, outliers were calculated separately for group and phase and excluded.^3^ All models were fitted with one between-subjects factor group (SAD, HC), one within-subject factor phase (1 to 7), and all possible interaction terms as fixed effects to measure the dependent variables heart rate (IBI), sympathetic arousal (SCL), and parasympathetic arousal (CDM). To adjust for possible baseline differences the last minute of the baseline-measurement was also entered into the mixed model as covariate. As this baseline measurement potentially influences the following measurements predominantly, statistics were calculated with Type-I ANOVA, entering the covariate first. Furthermore, intercepts for every participant were modeled as random effects.

For analyses of treatment effects on psychophysiological measures, mixed models were used once again. Outliers were calculated and excluded separately for groups, phase, and session^4^. Both models were fitted with one between-subjects factor group (CBT, WLC), two within-subject factors phase (1 to 7) and session (TSST-C_1, TSST-C_2), and all possible interaction terms as fixed effects again for dependent measures heart rate (IBI), sympathetic arousal (SCL), and parasympathetic arousal (CDM). Corresponding to the before treatment analyses, the last baseline minute was entered as covariate in the model and Type-I ANOVAs were calculated. Furthermore, intercepts for every participant were modeled as random effects.

The degrees of freedom for all mixed-model analyses were calculated with Satterthwaite approximation. Estimates for all models were optimized for the log-likelihood criterion. As debates about effect sizes in mixed models are still ongoing, no effect size could be reported (Rights & Sterba, 2019). However, the overall model quality was reported as marginal (only fixed effects) and conditional r-squares (fixed and random effects). Significant main effects and interactions were further analyzed (if relevant for the hypotheses) with post hoc t-tests for independent groups for the group comparisons and with t-tests for dependent groups for time and session comparisons. Cohen’s d effect sizes are reported for the post hoc tests.

## Results

All means and standard errors of the model for all phases are reported in the online supplements (S2).

### Before Treatment (First Study Aim)

#### Heart Rate (IBI)

For IBI, the mixed model resulted in a main effect for phase, F_(6,603)_ = 117.28, p < 0.001, but not for group, F_(1,99)_ = 2.75, p = 0.101. The interaction phase × group was significant, F_(6,603)_ = 4.57, p < 0.001. The covariate IBI_baseline_ showed a significant effect, F_(1,108)_ = 328.41, p < 0.001. The overall model explained R^2^ = 0.671 and R^2^ = 0.799, of the marginal and conditional variance, respectively.

**Fig. 3:**
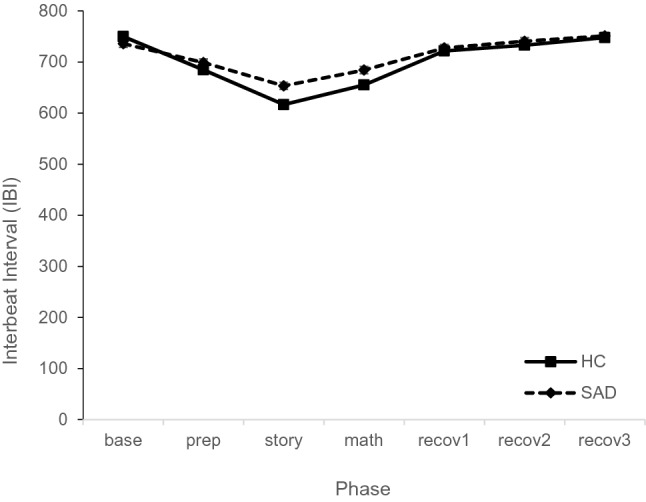
Interbeat interval during first TSST-C comparing SAD and HC group (estimated means and standard errors of the model)

Post-hoc tests to disentangle the interaction showed higher arousal in terms of lower IBI in the HC group compared to the SAD group in the phases story, t_(358.7)_ = 3.59, p < 0.001, d = 0.19, and math, t_(355.7)_ = 2.86, p = 0.004, d = 0.16 (see Fig. 3). There were no differences between groups in any of the other phases, ts < 1.39, ps > 0.165, ds < 0.08. Thus, children in the HC group showed a higher reactivity to the story than children in the SAD group.

#### Sympathetic Arousal: SCL

**Fig. 4:**
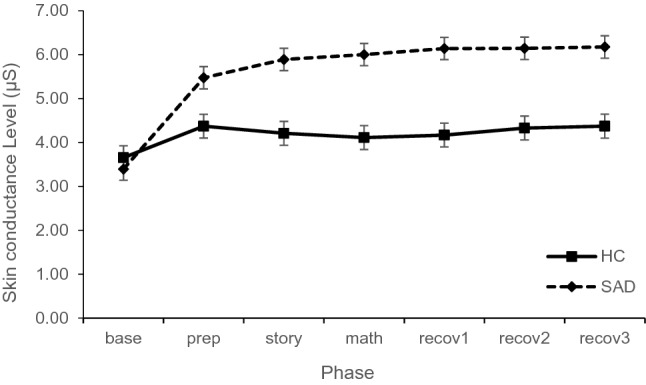
Skin conductance level during first TSST-C comparing SAD and HC group (estimated means and standard errors of the model)

For SCL, the mixed model resulted in a main effects for phase, F_(6,587)_ = 45.09, p < 0.001, and for group, F_(1,101)_ = 17.51, p < 0.001. Further, the interaction between phase × group reached significance, F_(6,587)_ = 19.03, p < 0.001 (see Fig. 4). The covariate SCL_baseline_ showed a significant effect, F(_1,104)_ = 400.33, p < 0.001. The overall model explained R^2^ = 0.800 and R^2^ = 0.950 of the marginal and conditional variance, respectively.

Post-hoc tests showed higher sympathetic arousal in the SAD group compared to the HC group in all phases, ts > 2.88, ps < 0.004, ds > 0.23, except for the first phase, t_(153.3)_ = 0.68, p = 0.496, d = 0.06. That is, children in the SAD group showed a tonic sympathetic hyperarousal throughout the stress and recovery phases. Therefore, even though starting out from a similar level, children in the SAD showed more sympathetic reactivity and were not able to recover to baseline as swiftly as children in the HC group.

#### Parasympathetic Arousal: CDM as Assessment of HRV

**Fig. 5:**
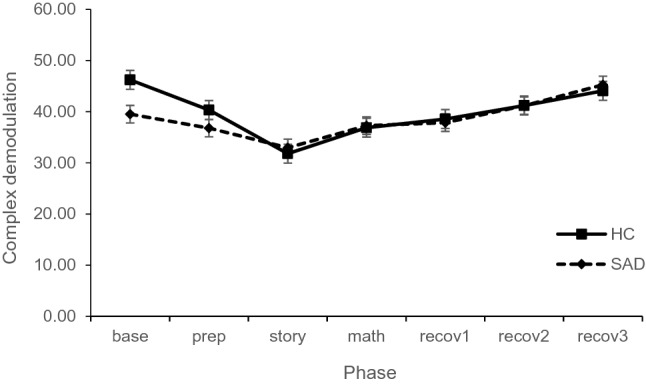
Parasympathetic arousal during first TSST-C comparing SAD and HC group (estimated means and standard errors of the model)

For CDM, the mixed model yielded a main effect for phase, F_(6,591)_ = 18.43, p < 0.001, but not for group, F_(1,99)_ = 0.43, p = 0.515, and an interaction effect phase × group, F_(6,591)_ = 2.34, p = 0.030 (see Fig. 5). The covariate CDM_baseline_ showed a significant effect, F(_1,109)_ = 143.28, p < 0.001. The overall model explained R^2^ = 0.450 and R^2^ = 0.682 of the marginal and conditional variance, respectively.

Post-hoc tests showed lower parasympathetic arousal in the SAD group compared to the HC group in the first phase, t_(333.67)_ = 2.65, p = 0.009, d = 0.14, while there were no further group differences in any other phase, ts < 1.41, ps > 0.159, ds < 0.08. These results point to a blunted reactivity in HRV in children with SAD starting from a higher activation but settling to similar scores as the HC group in the stress reactivity phase.

### Comparison of Psychophysiological Response Patterns to CBT (Second Study Aim)

#### Heart Rate (IBI)

For IBI after treatment, the mixed model showed a main effect for phase, as well as significant interactions for group × phase, and phase × session (see Table 3). The overall model explained R^2^ = 0.661 and R^2^ = 0.788 of the marginal and conditional variance, respectively. Based on our hypotheses, the group × session effect would have been of main interest. As this effect did not reach significance, no post-hoc analyses were conducted. Thus, no effect of treatment could be shown for HR.

**Table 3:** Statistical results on physiological arousal before and after intervention

Variable	*df*	*F*	*p*
*IBI*			
IBI_pre_	1, 247	322.13	< .001
Group	1, 40	0.12	.733
Phase	6, 524	85.36	< .001
Session	1, 537	2.77	.097
Group × phase	6, 524	2.39	.028
Group × session	1, 226	1.96	.162
Phase × session	6, 524	1.64	.135
Group × phase × session	6, 524	0.36	.907
*SCL*			
SCL_pre_	1, 513	1192.37	< .001
Group	1, 37	3.33	.076
Phase	6, 509	18.77	< .001
Session	1, 512	15.86	< .001
Group × phase	6, 509	0.90	.493
Group × session	1, 517	0.14	.708
Phase × session	6, 509	0.52	.790
Group × phase × session	6, 509	0.27	.950
*CDM*			
CDM_pre_	1, 258	71.79	< .001
Group	1, 35	0.06	.800
Phase	6, 514	11.55	< .001
Session	1, 527	4.39	.037
Group × phase	6, 514	1.19	.312
Group × session	1, 516	0.42	.517
Phase × session	6, 514	0.27	.953
Group × phase × session	6, 514	0.64	.696

*IBI* interbeat intervals as assessment of sympathetic and parasympathetic arousal, *SCL* skin conductance levels as assessment of electrodermal activity (sympathetic), *CDM* complex demodulation rate as assessment of HRV (parasympathetic)

#### Sympathetic Arousal: SCL

For SCL after treatment, there were main effects for SCL_baseline_, phase, and session (see Table 3 and online supplements for further discussion, S4). The overall model explained R^2^ = 0.771 and R^2^ = 0.878 of the marginal and conditional variance, respectively. As there were no effects concerning our hypotheses, no post-hoc analyses were conducted. Thus, no effect of treatment could be shown for SCL.

#### Parasympathetic Arousal: CDM as Assessment of HRV

For CDM after treatment, main effects occurred for CDM_baseline_, phase, and session (see Table 3 and online supplements for further discussion, S4). The overall model explained R^2^ = 0.270 and R^2^ = 0.557 of the marginal and conditional variance, respectively. No post-hoc analyses were conducted as there were no effects based on our hypotheses. Thus, no effect of treatment could be shown for HRV.

## Discussion

This study investigated psychophysiological arousal using measures of sympathetic and parasympathetic activity in children with SAD before and after CBT. Results before CBT are in line with previous studies (Asbrand et al., 2017; Schmitz et al., 2013; Siess et al., 2014). Children with SAD showed blunted reactivity in heart rate with elevated levels of HR in children with SAD compared to HC children during stress. Further, children with SAD had elevated levels of tonic sympathetic arousal as indexed by SCL compared to HC. Finally, children with SAD showed lower parasympathetic arousal during the baseline compared to HC children. The intervention as an experimental manipulation to investigate psychophysiological changes did not yield any significant results. Children with SAD receiving treatment did not differ from children in the waitlist condition in a repeated social stress test.

### Stability and Informative Value of the Autonomic Response in Child SAD

Interestingly, our results replicate and extend previous studies which used a social stress test but in a subclinical sample (Schmitz et al., 2013) or a smaller and slightly younger sample (Schmitz et al., 2011). Restricted autonomic flexibility seems to be a stable finding in children with SAD suggesting a lack of flexibility to swiftly adapt to changing environments (Beauchaine, 2001; Porges, 2007; Porges et al., 1996; Thayer & Lane, 2000).

Based on these results, we expected to induce changes using a treatment for SAD, i.e. an increase in flexibility after CBT. Nevertheless, while children in the treatment condition responded appropriately with a significant decrease in SAD symptoms (Asbrand et al., 2020), psychophysiological measures of autonomic flexibility did not change. In a post-hoc approach, potentially confounding factors such as age, body mass index and state anxiety (Kudielka et al., 2004) were examined but no significant effects concerning these covariates were found. While research on treatment effects on psychophysiology in anxiety disorders is scarce, research in adults (Mulkens et al., 2001) is in line with our findings. The authors report that patients with SAD report reduced fear of blushing after treatment which is, however, not paralleled by appropriate changes in physiological activation. Similarly, in an attempt to validate virtual reality treatment in anxiety symptoms and anxiety disorders, another study (Diemer et al., 2014) concludes that treatment effects of virtual reality on heart rate and on psychophysiological habituation remain inconclusive. Interestingly, other research points to a different perspective on psychophysiology. Instead of treating physiological variables as a possible outcome of therapy, one line of research argues that the focus on physiology might possibly serve as a potent treatment variable (Goessl et al., 2017). The authors report in a meta-analysis that HRV biofeedback, e.g. including mindfulness-based methods, is associated with reductions in self-reported stress and anxiety. The treatment mechanism is assumed to be related to emotion regulation or more specifically self-regulation which can be indexed with physiological measures, especially HRV. High HRV predicts self-regulatory strength and reduced negative affect during social stress, which is why HRV biofeedback seems a plausible intervention to reduce stress (Goessl et al., 2017).

Nevertheless, differences between children with SAD and HC children were apparent before treatment. Possibly, psychophysiological reactivity might be used as a diagnostic tool to predict successful therapy outcome (e.g. (Lang et al., 1998). For example, research suggests that patients showing higher physiological reactivity to stress are more likely to have a successful therapeutic experience (cf., (Lang et al., 2016). In line with the Research Domain Criteria Initiative (RDoC; (Kozak & Cuthbert, 2016), psychophysiological irregularities might be an important puzzle piece to understand disorders beyond DSM 5 categories (Lang et al., 2016). For example, patients’ ratings of emotional distress are similar while some can be classified as physiological hyper- and others as hypo-responders (Lang et al., 2016). Thus, the classification based subjective and additional biological factors could lead to the description of endophenotypes. Keeping in mind the low response rates in SAD (Hudson et al., 2015), these endophenotypes could provide useful for the improvement of treatment effects by providing endophenotype-based differential treatments. While this line of thought is crucial for future research, analyses in line with the RDoC idea were not possible in the current sample due to limited sample size. Further, a recent meta-analysis (Seddon et al., 2020) has shown the importance of variation in the single application of TSST-C regarding the outcomes. For autonomic markers (here focus on HR and HRV), they discussed that age, type of sample, duration of TSST-C, and number of judges would influence physiology. That is, children show greater HR reactivity if age is examined dichotomously. This is in line with findings that both HR and HRV reactivity to stress decreases with age (Seddon et al., 2020). Also, physiological responses are stronger if the TSST-C takes longer (comparing 6 to 25 min) and more judges are present. Clinical samples have been shown—similar to previous findings on social anxiety (Schmitz et al., 2013)—to show a lower HR reactivity. The current study was very homogeneous and conservative regarding these factors, examining a short span of age (9–13 years), well examined groups (SAD vs. HC), and the original set-up of the TSST-C (two judges, 10 min of stress; Buske-Kirschbaum et al., 1997). However, a variation of the set-up might lead to different outcomes, especially concerning effects of CBT. It might be interesting to compare CBT effects of a more stressful TSST-C (i.e. more judges, longer duration) against a less stressful TSST-C (i.e. max. two judges, shorter duration max. 8 min). It can be argued that a more stressful TSST-C brings out clearer changes in the CBT group as they have more possibility to regulate their arousal. However, it could also be assumed that a less stressful TSST-C is more in line with CBT itself and thus a better assessment of CBT changes. As this is the first study overall regarding CBT effects, current results can only instigate future lines of research. Further, while moderators are very helpful in understanding risk factors for certain groups (e.g. age), it is unclear if CBT effects are also impacted by the same moderators. In fact, this may not be likely, as the moderators identified in the meta-analyses related to physiological levels at one given assessment point. The CBT effects under investigation here, however, related to differences between two groups across assessment points. Potential moderators apply similarly at both assessments and are therefore less likely to explain the (lack of) intervention effects. Only if moderators would interact with the intervention conditions, they may evolve as potential explanations.

### Limitations, Strengths and Implications

While the study was planned and conducted carefully, several limitations apply. First, the HC group participated only in one TSST-C as the project focused on longitudinal effects in SAD. However, a comparison between normative changes in the HC group to potential treatment changes in the SAD group would be an interesting angle for future studies. Second, psychophysiological studies include the possibility to focus on a variety of sympathetic and parasympathetic markers. The current study included a wide set including IBI, SCL, and CDM while reporting t-wave amplitude and RMSSD in a secondary manner in online supplements (S3). Still, even though the parameters were carefully chosen to cover a range of autonomic markers, a different subset might have led to different results. Further, TSST reactivity is dependent on a wide set of moderators (e.g., duration, age, number of judges; Seddon et al., 2020). While we opted for the often-used original version with a very homogenous sample (e.g. balanced gender, limited age group from 9 to 13 years), it might be possible that a different sample (e.g. adolescents instead of children) leads to different findings. Finally, the nature of the stress tasks used in the highly standardized stress test (TSST-C) are not equally „social “in nature: the speech task likely arouses more SAD-concerns than the math task. While the latter might be stressful (performing a math task in front of others), this task may be not as appropriate to elicit SAD-typical concerns. The differential social relevance of the task in the TSST-C may therefore contribute to the lack of change in physiology across assessments. Future research may therefore weigh the cost and the benefits of using a well-investigated standardized paradigm versus a higher suitability for the specific sample under investigation (task-sample match). Group differences before treatment, and lack of group or interaction effects after treatment might point to the fact that the treatment was either not long enough or did not include the right components to tackle changes in physiological markers. However, treatment did show an improvement of social anxiety symptoms (Asbrand et al., 2020) and cognitions (Asbrand et al., 2019). The current treatment focused on the gold-standard of psychoeducation, cognitive restructuring, skills training and exposure. Future studies might include relaxation or mindfulness based methods as these seem to be more strongly related to physiological arousal (Goessl et al., 2017). However, it cannot be ruled out that psychophysiological arousal might be an epiphenomenon instead of an etiological marker of SAD. While there are now several results supporting the notion of dysregulated autonomic activity in childhood SAD (Schmitz et al., 2011, 2013), more studies are needed to clarify changes by treatment. Finally, the possibility of physiology as a component of an endophenotype based on RDoC criteria to predict treatment response should be targeted in future studies. This also leads to the important next step of exploring interdependence of different factors (e.g., cognitive, behavioral, physiological) and extend our understanding of social anxiety by describing underlying patterns of endophenotypes.

In conclusion, the present study (1) supports previous findings in identifying psychophysiological differences (namely, blunted HR reactivity, tonic hyperarousal and parasympathetic differences during baseline) between children with SAD and HC; and (2) could not find any significant effect of a standard comprehensive CBT intervention on the psychophysiological levels. While there are several potential explanations for this finding (e.g. physiological changes may take longer than cognitive changes; or suitability of the intervention for inducing psychophysiological changes limited), only studies with a longitudinal design targeting both, cognition and physiology will be able to elucidate potential mechanisms further.

## Supplementary Information

Below is the link to the electronic supplementary material.

Electronic supplementary material 1 (DOCX 38 kb)

## Abbreviations

ANS: Autonomic nervous system
Bpm: Beats per minute
CBT: Cognitive behavior therapy
CDM: Complex demodulation rate
DGPs: German Society for Psychology
HC: Healthy control
HR: Heart rate
HRV: Heart rate variability
Hz: Hertz
IBI: Inter-beat interval
Kinder-DIPS: Diagnostic Interview for Mental Disorders in Children and Adolescents
PNS: Parasympathetic nervous system
RCT: Randomized controlled trial
RDoC: Research Domain Criteria Initiative
SAD: Social anxiety disorder
SCL: Skin conductance level
SNS: Sympathetic nervous system
SPAI-C: Social Phobia and Anxiety Inventory for Children
RMSSD: Root Mean Square of the Successive Differences
TSST-C: Trier Social Stress Test for Children
WLC: Waitlist control

## Footnotes

1 Further results are reported elsewhere (treatment outcome, Asbrand et al., 2020; changes in post-event processing based on treatment, Asbrand et al., 2019; stability of the cortisol response despite treatment, Asbrand et al., 2019 perception of physiological arousal, Asbrand et al., 2020) or are being prepared for submission (social performance). To ensure maximal transparency, all articles include cross-references to other reports on measures used to investigate potential treatment-related effects.

2 Treatment site was not entered in the analysis model as no differences appeared in a first comparison of sites.

3 Statistical outliers were identified as 2.5 *SD*s above or below the mean. The proportions of outliers for all analyses were below 5%.
