# Supplementary material for: Autonomic Dysregulation in Child Social Anxiety Disorder: An Experimental Design Using CBT Treatment
Source: Appl Psychophysiol Biofeedback. 2022 Jun 1;47(3):199–212. doi: 10.1007/s10484-022-09548-0 (PMC9296402; doi:10.1007/s10484-022-09548-0)
Supplement: Supplementary file 1 — Electronic supplementary material 1 (DOCX 38 kb) [file 10484_2022_9548_MOESM1_ESM.docx]

**Supplements**

# **S1. Stories**

*Version A*

Yesterday my best friend Robert and I were walking home from school.

Suddenly, we had the idea to visit Mr. Grimme who lives in the big old house that is in the dark forest near our town.

Mr. Grimme was a crazy old man and our parents did not like the idea of us visiting him. There was a rumor in town that there was a secret to the old house.

When we got there, we were surprised to see that the door was open. Suddenly, we heard a strange noise and cautiously we entered the dark hallway....

*Version B*

Yesterday my best friend Robert and I were walking home from school.

Suddenly, we had the idea to walk to the old, dilapidated quarry, which is located in the dark forest near our town.

No work had been done in the quarry for a long time and it was actually not allowed to enter it. Our parents did not like the idea of us exploring the quarry because people told strange stories about it.

When we got there, we were surprised to see that the gate in the fence around the quarry was open. Suddenly, we heard a strange noise and cautiously we entered the quarry....

# **S2. Means and standard deviations of reported measures**

## **Before treatment**

Table S1.

*Means and standard errors IBI, SCL and CDM before treatment.*

|  | M(SD) | | | | | | | | | | | | | |
| --- | --- | --- | --- | --- | --- | --- | --- | --- | --- | --- | --- | --- | --- | --- |
|  | Base | | Prep | | Speech | | Math | | Recov1 | | Recov2 | | Recov3 | |
|  | SAD | HC | SAD | HC | SAD | HC | SAD | HC | SAD | HC | SAD | HC | SAD | HC |
| IBI | 735.66 (6.94) | 749.68 (7.41) | 698.81 (6.94) | 684.59 (7.47) | 653.27 (6.93) | 616.53 (7.46) | 684.14 (6.88) | 654.95 (7.46) | 727.30 (6.94) | 721.59 (7.46) | 740.48 (6.94) | 732.74 (7.46) | 750.94 (6.94) | 747.66 (7.46) |
| SCL | 3.40 (0.25) | 3.66 (0.27) | 5.48 (0.25) | 4.37 (0.27) | 5.90 (0.25) | 4.21 (0.27) | 6.00 (0.25) | 4.11 (0.27) | 6.14 (0.25) | 4.17 (0.27) | 6.15 (0.26) | 4.33 (0.27) | 6.18 (0.26) | 4.37 (0.27) |
| CDM | 39.52 (1.72) | 46.21 (1.84) | 36.78 (1.84) | 40.33 (1.70) | 32.95 (1.84) | 31.79 (1.69) | 37.27 (1.70) | 36.88 (1.84) | 37.85 (1.70) | 38.57 (1.84) | 41.18 (1.70) | 41.21 (1.84) | 45.25 (1.69) | 44.07 (1.84) |

## **After treatment**

Table S2.

*Means and standard errors IBI, SCL and CDM before and after treatment.*

|  |  | M(SD) | | | | | | | | | | | | | |
| --- | --- | --- | --- | --- | --- | --- | --- | --- | --- | --- | --- | --- | --- | --- | --- |
|  |  | Base | | Prep | | Speech | | Math | | Recov1 | | Recov2 | | Recov3 | |
|  |  | CBT | WLC | CBT | WLC | CBT | WLC | CBT | WLC | CBT | WLC | CBT | WLC | CBT | WLC |
| TSST-C 1 | IBI | 718.07 (10.51) | 724.94 (12.80) | 681.69 (10.51) | 682.0 (12.80) | 630.01 (10.51) | 618.02 (12.56) | 651.70 (10.51) | 665.66 (12.56) | 699.81 (10.51) | 723.60 (12.80) | 715.81 (10.51) | 731.96 (12.80) | 728.19 (10.51) | 738.92 (12.80) |
|  | SCL | 4.15 (0.44) | 4.07 (0.51) | 6.23 (0.44) | 5.81 (0.51) | 6.62 (0.44) | 5.80 (0.51) | 6.82 (0.43) | 5.66 (0.51) | 7.05 (0.43) | 5.64 (0.51) | 6.93 (0.44) | 5.71 (0.51) | 6.95 (0.44) | 5.71 (0.51) |
|  | CDM | 39.59 (2.47) | 38.38 (3.00) | 34.73 (2.47) | 38.00 (3.00) | 30.80 (2.47) | 30.09 (2.94) | 33.44 (2.47) | 37.89 (3.00) | 34.90 (2.51) | 36.47 (3.00) | 39.47 (2.51) | 37.46 (3.00) | 45.66 (2.47) | 39.43 (3.00) |
| TSST-C 2 | IBI | 718.20 (10.53) | 716.17 (12.76) | 663.88 (10.68) | 635.89 (12.76) | 327.55 (10.53) | 605.66 (12.49) | 660.87 (10.53) | 657.9 (12.49) | 704.88 (10.53) | 719.56 (12.49) | 717.37 (10.53) | 730.87 (12.49) | 725.50 (10.53) | 743.43 (12.49) |
|  | SCL | 4.13 (0.43) | 3.71 (0.51) | 5.87 (0.43) | 5.03 (0.52) | 5.82 (0.43) | 4.99 (0.52) | 5.79 (0.43) | 5.01 (0.52) | 6.23 (0.43) | 5.16 (0.52) | 6.39 (0.43) | 5.47 (0.52) | 6.44 (0.43) | 5.64 (0.52) |
|  | CDM | 38.70 (2.47) | 37.30 (3.00) | 33.0 (2.47) | 31.78 (3.00) | 31.34 (2.47) | 27.43 (3.00) | 33.25 (2.47) | 35.63 (3.00) | 36.05 (2.47) | 33.57 (3.00) | 36.96 (2.47) | 36.68 (3.00) | 40.92 (2.47) | 39.63 (3.00) |

# **S3. Further sympathetic and parasympathetic measures**

## **Before Treatment**

### *Sympathetic arousal: TWA*

For TWA, the mixed model resulted in a significant main effect phase, *F*_(6,604)_ = 27.98, *p* < .001, but not group, *F*_(1,102)_ = 0.15, *p* = .695. Further, no significant interaction effect phase × group was found, *F*_(6,604)_ = 1.11, *p* = .354 (see Figure S1). The covariate TWA_baseline_ showed a significant effect, *F*_(1,104)_ = 989.35, *p* < .001. The overall model resulted in an explained variance of marginal *R*^2^ = .833 and conditional *R*^2^ = .897, resp. As no effect relevant for the hypotheses was found, no post-hoc tests were conducted.

Figure S1. T-wave amplitude during first TSST-C comparing SAD and HC group (estimated means and standard errors of the model).

### *Parasympathetic arousal: RMSSD*

For RMSSD, the mixed model resulted in a significant main effect phase, *F*_(6,574)_ = 36.61, *p* < .001, but not group, *F*_(1,87)_ = 0.31, *p* = .579. Further, only a trend for a significant interaction effect phase × group was found, *F*_(6,574)_ = 1.94, *p* = .072 (see Figure S2). The covariate RMSSD_baseline_ showed a significant effect, *F*_(1,98)_ = 163.16, *p* < .001. The overall model resulted in an explained variance of marginal *R*^2^ = .499 and conditional *R*^2^ = .735, resp. As no effect relevant for the hypotheses was found, no post-hoc tests were conducted.

Figure S2. RMSSD during first TSST-C comparing SAD and HC group (estimated means and standard errors of the model)..

Table S3.

*Means and standard errors TWA and RMSSD.*

|  | M(SD) | | | | | | | | | | | | | |
| --- | --- | --- | --- | --- | --- | --- | --- | --- | --- | --- | --- | --- | --- | --- |
|  | Base | | Prep | | Speech | | Math | | Recov1 | | Recov2 | | Recov3 | |
|  | SAD | HC | SAD | HC | SAD | HC | SAD | HC | SAD | HC | SAD | HC | SAD | HC |
| TWA | 294.11 (7.84) | 303.86 (8.33) | 271.64 (8.33) | 285.67 (7.95) | 251.34 (7.89) | 240.44 (8.39) | 262.44 (7.90) | 259.13 (8.39) | 306.68 (7.90) | 305.14 (8.33) | 308.49 (7.90) | 309.09 (8.33) | 284.46 (7.90) | 298.03 (8.33) |
| RMSSD | 47.70 (2.32) | 55.79 (2.51) | 45.81 (2.30) | 49.05 (2.53) | 37.22 (2.30) | 34.66 (2.53) | 41.09 (2.32) | 40.88 (2.53) | 47.92 (2.32) | 49.82 (2.53) | 53.74 (2.32) | 53.47 (2.53) | 58.14 (2.30) | 57.88 (2.53) |

## **Treatment effects**

### *Sympathetic arousal: TWA*

For TWA after treatment, significant main effects were found for TWA_baseline_, and phase (see Table S1). The overall model resulted in an explained variance of marginal *R*^2^ = .823 and conditional *R*^2^ = .875, resp. As no effects of interest for the hypotheses appeared, no post-hoc analyses were conducted.

### *Parasympathetic arousal: RMSSD*

For RMSSD after treatment, significant main effects were found for RMSSD_baseline_, , and phase (see Table S4). The overall model resulted in an explained variance of marginal *R*^2^ = .430 and conditional *R*^2^ = .624, resp. As no effects of interest for the hypotheses appeared, no post-hoc analyses were conducted.

Table S4.

*Results on physiological arousal before and after intervention.*

| Variable | *df* | *F* | *p* |
| --- | --- | --- | --- |
| **TWA**  TWA_pre_  Group  Phase  Session  Group × Phase  Group × Session  Phase × Session  Group × Phase × Session | 1, 136  1, 41  6, 511  1, 541  6, 511  1, 530  6, 511  6, 511 | 881.02  0.27  24.21  0.02  1.09  0.16  0.85  0.80 | <.001  .608  <.001  .886  .366  .691  .532  .572 |
| **RMSSD**  RMSSD_pre_  Group  Phase  Session  Group × Phase  Group × Session  Phase × Session  Group × Phase × Session | 1, 176  1, 38  6, 515  1, 529  6, 515.  1, 517  6, 515  6, 515 | 133.13  1.22  23.23  1.21  0.99  1.31  0.65  0.86 | <.001  .277  <.001  .273  .429  .254  .690  .523 |

Table S5.

*Means and standard errors TWA and RMSSD before and after treatment.*

|  |  | M(SD) | | | | | | | | | | | | | |
| --- | --- | --- | --- | --- | --- | --- | --- | --- | --- | --- | --- | --- | --- | --- | --- |
|  |  | Base | | Prep | | Speech | | Math | | Recov1 | | Recov2 | | Recov3 | |
|  |  | CBT | WLC | CBT | WLC | CBT | WLC | CBT | WLC | CBT | WLC | CBT | WLC | CBT | WLC |
| TSST-C 1 | TWA | 265.98 (12.05) | 260.90 (14.67) | 246.92 (12.05) | 230.40 (15.42) | 213.36 (12.05) | 202.3 (15.05) | 222.50 (12.05) | 228.79 (15.00) | 276.11 (12.05) | 281.60 (15.00) | 286.48 (12.05) | 276.65 (15.00) | 262.49 (12.05) | 241.11 (15.00) |
|  | RMSSD | 45.64 (3.19) | 44.77 (3.83) | 42.68 (3.19) | 44.33 (3.83) | 35.13 (3.14) | 30.92 (3.75) | 35.98 (3.14) | 39.95 (3.83) | 44.50 (3.19) | 43.56 (3.83) | 53.13 (3.14) | 47.42 (3.83) | 58.36 (3.14) | 49.50 (3.83) |
| TSST-C 2 | TWA | 256.68 (12.31) | 258.26 (14.61) | 233.56 (12.31) | 207.54 (14.26) | 222.54 (12.31) | 191.36 (14.26) | 235.45 (12.31) | 226.19 (14.26) | 276.52 (12.31) | 286.21 (14.26) | 266.36 (12.31) | 289.65 (14.26) | 265.65 (12.31) | 270.07 (14.26) |
|  | RMSSD | 47.40 (3.14) | 44.26 (3.83) | 41.24 (3.19) | 34.67 (3.83) | 37.08 (3.14) | 27.99 (3.74) | 37.75 (3.14) | 37.54 (3.74) | 49.28 (3.14) | 41.67 (3.82) | 49.94 (3.14) | 46.02 (3.82) | 52.98 (3.19) | 51.25 (3.82) |

# **S4. Main effects of sympathetic and parasympathetic arousal**

Interestingly, both SCL as a sympathetic measure as well as CDM as a parasympathetic measure show a significant effect of session, i.e. changes in arousal irrespective of treatment. This might be caused by the fact that children knew what to expect in the second task (i.e. some sort of habituation effect). However, the effects are not strong enough to provide a significant effect of heart rate (innervated by both sympathetic and parasympathetic nerves) as well as other measures (see supplements with non-significant effects for T-Wave Amplitude and RMSSD). These inconsistent findings across parameters (i.e. have two out of four parameters of sympathetic and parasympathetic parameters) are difficult to interpret. While it is possible to conclude that these main effects on SCL and CDM may reflect habituation (as it is related to the second repeated assessment and not the specific condition). However, this does not explain why other parameters also elicited by or associated with sympathetic and parasympathetic activity do not reflect this effect. Therefore, this (potential) habituation effect warrants further research.
